# Oxidative stress model of lipopolysaccharide combined with thrombin inducing in broiler chicks

**DOI:** 10.3389/fvets.2024.1369515

**Published:** 2024-11-18

**Authors:** Huiyu Shi, Fengyuan Yang, Zeru Peng, Junlong Wu, Qin Wang, Pingfei Qiu, Ruiying Bao, Liangmin Huang, Xiaochun Li, Haiwen Zhang, Xuemei Wang

**Affiliations:** Department of Animal Science and Technology, School of Tropical Agriculture and Forestry, Hainan University, Haikou, China

**Keywords:** LPS, thrombin, AA broiler, immune indexes, oxidative stress

## Abstract

Lipopolysaccharides (LPS) are commonly used to construct inflammation models. However, poultry have a certain degree of tolerance to LPS due to the lack of thrombin XI and XII in their bodies. Thrombin activation produces clotting factors that can cleave prothrombin to form thrombin. The purpose of this study was to construct a chick oxidative stress model used different concentrations of LPS combined with thrombin in order to screen for the optimal concentration for constructing the oxidative stress model, and to explore the effects of this stimulus on various indicators of chicks. Eighty-one young chicks (4-days-old) were randomly divided into three groups with 27 chicks per group where each group contained 3 replicates with 9 birds each: a control group (physiological saline), a low-dose group (LPS 5 mg/kg thrombin 150 U/kg), and a high-dose group (LPS 10 mg/kg thrombin 300 U/kg). The results indicated that compared with the control group, the low-dose group and the high-dose group significantly increased the content of Malondialdehyde (MDA) in serum and reduced the content of T-AOC, GSH-PX and SOD, respectively. Meanwhile, the levels of NO and inflammatory factors IL-1β, IL-6 and TNF-α TNF-α in the liver were significantly increased in the low-dose and high-dose groups compared with the control group, respectively. Liver and thymus tissue sections from the low- and high-dose groups showed hemorrhage, hemolysis, and a small amount of exudation. In terms of inflammatory effect, the serum MDA content and the levels of NO, IL-1β, IL-6 and TNF-α factors in the liver were significantly increased in the low-dose group compared with the high-dose group. On histopathological observation, tissue damage was more pronounced in the low-dose group than in the high-dose group. In conclusion, LPS combined with thrombin could induce oxidative stress in chicks and the pathological changes of the low-dose effect are more pronounced.

## 1 Introduction

The investigation of oxidative stress represents a significant area of inquiry in the context of poultry, particularly concerning the development of inflammatory models tailored for broiler chicks. Oxidative stress denotes a disruption in the equilibrium between the generation of free radicals and the organism’s capacity to counteract these reactive oxygen species. Inflammation serves as the principal effector mechanism of the innate immune system, functioning to eradicate the initiating stimulus, facilitate tissue repair, and restore physiological equilibrium (1). In experimental modelling of inflammation, lipopolysaccharides (LPS) are frequently employed due to their ability to elicit a robust inflammatory response. LPS administration can incite activation of the immune system within the gastrointestinal milieu, potentially culminating in systemic dissemination and subsequent endotoxemia (2).

LPS, a constituent found in the cell walls of Gram-negative bacteria, serves as a potent initiator of innate immune responses, orchestrating the recruitment of inflammatory cells to the affected tissue and precipitating a cascade of reactions encompassing endotoxin shock, sepsis, and immune reactions (3). Notably, LPS also instigates endothelial responses typified by the upregulation of cytokines, adhesion molecules, and tissue factors (4). Pathophysiologically, immune dysregulation induced by LPS is characterized by perturbations in microvascular endothelial permeability to macromolecules (5). Moreover, endothelial cells respond to LPS by undergoing morphological alterations, fostering intercellular gap formation, and heightening transendothelial permeability (6). Nonetheless, avian species display a degree of resistance to LPS owing to the absence of clotting factors XI and XII within their physiological milieu (7).

Beyond its pivotal role as the ultimate protease orchestrating the coagulation cascade, thrombin assumes significant prominence as a potent mitogenic factor. Thrombin, generated through the activation of clotting factors, cleaves prothrombin to yield thrombin, thereby augmenting the inflammatory response (8). The concept of intricate interplay between inflammation and coagulation has garnered substantial recognition in recent years (9). Vascular inflammation instigates activation of the coagulation pathway, while reciprocally, coagulation exerts a profound influence on the inflammatory milieu (10). Notably, the activation of the coagulation cascade, marked by thrombin generation as a pivotal protease, fosters a proinflammatory environment that particularly impacts endothelial function and innate immune cell behavior (11). Thrombin executes its diverse functions, including various proinflammatory vascular responses, primarily through the activation of protease-activated receptors (PARs) (12). Upon injury to the endothelial layer of blood vessels, the activation of the coagulation cascade becomes integral to the natural healing process. Thrombin, generated at the surface of activated platelets, amplifies the coagulation cascade and facilitates additional platelet recruitment to the developing thrombus. Concurrently, activated platelets synthesize and release a myriad of proinflammatory and immunomodulatory molecules, thus contributing to both inflammatory and reparative processes (13).

There is limited research on the optimal concentration of LPS and thrombin for building an oxidative stress model in chicks. The study’s objectives are to evaluate the inflammatory response to different LPS and thrombin concentrations, assess the effect of LPS and thrombin on immune function, and investigate the inflammatory response mechanisms induced by LPS and thrombin in chicks. By identifying the optimal concentration of LPS and thrombin for building an oxidative stress model in chicks, this study aims to contribute to the development of a more effective model for studying the oxidative stress response in poultry and the potential treatment of inflammatory diseases.

## 2 Materials and methods

### 2.1 Main reagents

The following chemical reagents were used in this study: injectable white-browed viper venom thrombin (produced by Jinzhou Hong Pharmaceutical Co., Ltd.), LPS (produced by Beijing Solabao Technology Co., Ltd.), HE staining solution (produced by Biosharp), NO assay kit (A013-2-1), T-SOD assay kit (A001-1-2), MDA test kit (A003-1-2), and T-AOC test kit (A015-1-2)(produced by Nanjing Jiancheng Bioengineering Institute, Nanjing, China), chick IL-10 enzyme-linked immunosorbent assay kit (ML-14), chick IL-6 enzyme-linked immunosorbent assay kit (ML-13), chick IL-1β enzyme-linked immunosorbent assay kit (ML-12), and chick TNF-α attachment assay kit (ML-11) (produced by Hailian Biological Engineering Co., Ltd., Shanghai, China).

### 2.2 Experimental animals

We obtained 81 healthy SPF-level AA broiler chicks (4-days-old) with an almost equal sex ratio by incubating 150 SPF-level AA broiler chick eggs. The chicks were conventionally reared in the laboratory facility, housed in cages throughout the experiment, and fed with a basic diet. The chicks were allowed to feed and drink freely during the experiment. We ensured regular cleaning and disinfection of the experimental chick house to maintain hygiene. Commercial broiler chick feed was used in this experiment.

### 2.3 Experimental design and animal care

Eighty-one healthy SPF-level AA broiler chicks were randomly allocated into three treatment groups: the control group (normal saline), low-dose group (LPS 5 mg/kg + thrombin 150 U/kg), and high-dose group (LPS 10 mg/kg + thrombin 300 U/kg). Each group was replicated three times, with nine chicks in each replicate, and the experiment was conducted for a period of 7 days. The chicks in the control group were injected with 0.2 mL of sterile physiological saline, while those in the other groups were injected with 0.2 mL of the corresponding solution at different concentrations. Thrombin was injected subcutaneously into the neck, and LPS was injected intraperitoneally. The chicks were housed in a conventional laboratory facility, with cages providing 24-h light and temperature control. They were provided with *ad libitum* access to feed and water, and the experimental chick house was cleaned and disinfected regularly.

### 2.4 Growth performance and mortality rate

To measure growth performance and mortality rate, the chicks were fasted and weighed by replicate. Weight gain was recorded, and the amount of feed consumed by each group was weighed to calculate daily weight gain, daily feed intake, and feed-to-meat ratio. The health status and mortality of the animals were observed and recorded throughout the experiment. The average daily weight gain (g/d) was calculated as follows: (average final weight − average initial weight)/number of days in the experiment. Feed conversion ratio was calculated as daily feed intake (g)/daily weight gain (g).

### 2.5 Clinical symptom evaluation

To evaluate clinical symptoms, the mental status, feathers, feeding, water intake, defecation, and other conditions of the chicks in each group were observed and recorded in real-time within 24 h after injection. Clinical symptoms were evaluated based on the observations.

### 2.6 Evaluation of temperature changes

To evaluate temperature changes, the thermometer was cleaned with 75% ethanol after drug injection. The temperature of each chick was measured for 2–3 min at 1, 3, 6, 12, and 24 h after injection. The thermometer was left to stabilize, and the average temperature at each time point was recorded and compared with the baseline temperature (0 h).

### 2.7 Measurement of serum indexes

To determine the impact of the drug on the serum indices, 8 randomly selected chicks from each group were subjected to cardiac puncture and 0.5 mL of blood was collected at 0 h, 6 h, 12 h, 24 h, 3 days, and 7 days after injection. The collected blood was allowed to stand in a sterilized EP tube for 1–2 h, then centrifuged at 2,500 rpm for 15–20 min at 4°C. The upper serum was carefully collected and stored at −20°C for subsequent analysis.

The contents of various serum indices, including MDA, T-SOD, T-AOC, GSH-PX, and others, were measured to assess the impact of the drug on antioxidant indexes of the chicks.

### 2.8 Tissue pathology and observation of intestinal mucosal status

At injection 6 h and 5 days, select one chicken for each replicate of each group. Tissue samples of the liver, spleen, thymus, duodenum, jejunum, and ileum were collected under sterile conditions. Tissue paraffin sections were prepared using HE staining to observe the pathological conditions of each tissue and the status of the intestinal mucosa.

### 2.9 Measurement of inflammatory cytokine TNF-α, IL-1β, and IL-6

At injection 12 h, select one chicken for each replicate of each group. The liver tissue was subjected to a pre-chilled PBS (0.01 M, pH = 7.4) wash to eliminate residual blood and connective tissue. After weighing and mincing, PBS was added to the liver tissue in an appropriate volume (1:9 mass/volume ratio). The mixture was then homogenized and centrifuged at 12,000 r/min for 10 min. The supernatant was collected and stored at −20°C for further ELISA assay to evaluate the levels of inflammatory cytokines, namely TNF-α, IL-1β, IL-6, and IL-8. In case of any precipitate formation during storage, re-centrifugation was carried out prior to use.

### 2.10 Statistical analysis

All data are presented as the average of the pooled SEM values. Statistical analysis was performed using SPSS 26.0 for Windows (SPSS Inc., Chicago, IL, United States). GraphPad Prism 9.0.0 (121) software was used to make pictures and statistical analysis. Between-group differences was assessed by the Shapiro–Wilk test of normal distribution followed by one-way ANOVA and Tukey’s test. A *p*-value less than 0.05 was considered statistically significant.

## 3 Results

### 3.1 Effect of LPS combined with thrombin on temperature in chick oxidative stress model

The results showed a significant change in rectal temperature at different time points after the injection of LPS and thrombin. In high-dose group compared to the control group, there was a significant decrease in temperature at 1 h after injection, followed by a significant increase at 3 h (*p* < 0.05). In low-dose group temperature increased significantly (*p* < 0.05) after 3 h after injection. At 24 h, the low dose group returned to normal temperature, while the high dose group showed a sustained increase in temperature (*p* < 0.05) (Table 1).

**Table 1:** Effect of LPS combined with thrombin on rectal temperature in chick oxidative stress model (°C).

Effect hours	Groups		
	Control	Low-dose	High-dose
0 h	40.49 ± 0.43^a^	40.5 ± 0.27^a^	40.27 ± 0.20^a^
1 h	40.70 ± 0.39^a^	40.57 ± 0.43^a^	40.22 ± 0.57^b^
3 h	40.36 ± 0.28^c^	41.02 ± 0.39^a^	40.83 ± 0.39^b^
6 h	40.05 ± 0.35^b^	41.04 ± 0.48^a^	40.90 ± 0.41^a^
12 h	40.52 ± 0.35^b^	41.37 ± 0.20^a^	41.14 ± 0.49^a^
18 h	39.90 ± 0.46^b^	39.99 ± 0.29^ab^	40.29 ± 0.44^a^
24 h	40.77 ± 0.56^b^	40.44 ± 0.49^b^	41.00 ± 0.24^a^

Different letters in the same row indicate significant differences (*p* < 0.05), while the same letters or no shoulder marks indicate no significant differences (*p* > 0.05).

### 3.2 Effects of LPS combined with thrombin on growth performance of chick oxidative stress model

It has been found that no significant difference in the average initial body weight of each treatment group (*p* > 0.05). However, compared with the control group, the daily weight gain of the test groups after LPS and thrombin injection showed a significant downward trend (*p* < 0.05). Among them, the high-dose group had the lowest daily weight gain (*p* < 0.05).

Moreover, the feed conversion ratio of the control group, low-dose group, and high-dose group were 1.75, 1.96, and 2.64, respectively, with a significant difference between the groups (*p* < 0.05) (Table 2). The study suggests that LPS may cause intestinal injury, which could lead to a reduction in feed conversion ratio, ultimately impacting the growth performance of chicks. There were no chick deaths during the trial.

**Table 2:** Effects of LPS combined with thrombin on growth performance of chick oxidative stress model.

Growth performance	Groups		
	Control	Low-dose	High-dose
Initial weight (g)	55.40 ± 3.32^a^	57.84 ± 2.69^a^	56.94 ± 3.17^a^
Final weight (g)	98.15 ± 2.29^a^	96.20 ± 1.10^b^	86.12 ± 1.57^c^
Daily gain weight (g)	6.11 ± 0.05^a^	5.48 ± 0.02^b^	4.17 ± 0.08^c^
Daily feed intake (g)	10.67 ± 0.09^a^	10.71 ± 0.06^a^	11.00 ± 0.22^a^
Feed conversion ratio	1.75 ± 0.02^c^	1.96 ± 0.01^b^	2.64 ± 0.05^a^

Different letters in the same row indicate significant differences (*p* < 0.05), while the same letters or no shoulder marks indicate no significant differences (*p* > 0.05).

### 3.3 Effect of LPS combined with thrombin on serum antioxidant indexes of chick oxidative stress model

**Figure 1:**
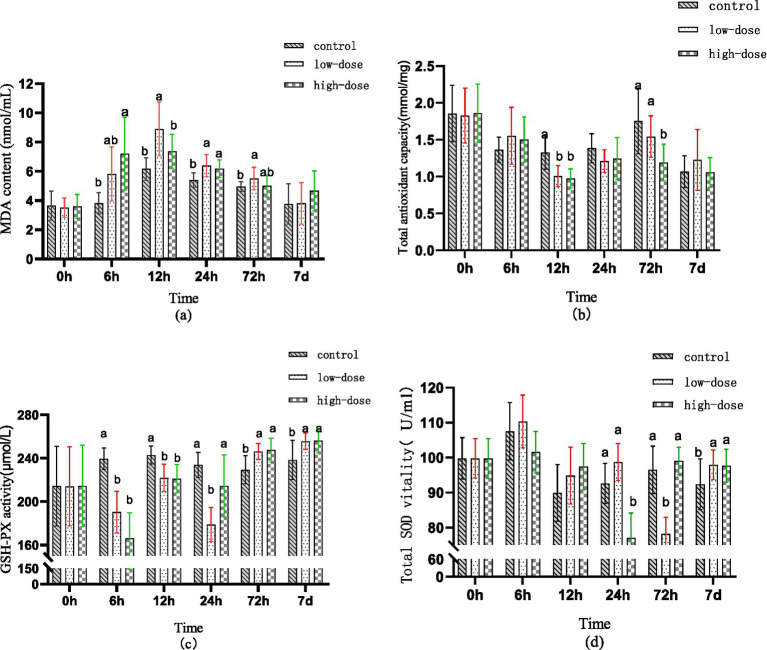
Effect of LPS combined with thrombin on serum antioxidant indexes of chick oxidative stress model at different time (a) MDA content (b) T-AOC (c) GSH-PX (d) SOD.

Figure 1a displays the variation in serum MDA levels during different time intervals of the entire test cycle. Compared to the control group, the low-dose group demonstrated a non-significant rise in serum MDA levels (*p* > 0.05) after 6 h of administration, while the high-dose group showed a significant increase in MDA levels (*p* < 0.05). After 12 h of administration, the low-dose group exhibited a significant increase in MDA levels (*p* < 0.05), while the high-dose group’s MDA levels continued to increase. Following 24 h of administration, both the low-dose and high-dose groups’ MDA levels remained elevated (*p* < 0.05). After 72 h of administration, the high-dose group’s MDA levels returned to normal, while the low-dose group’s MDA levels remained high (*p* < 0.05).

Figure 1b illustrates the changes in serum T-AOC at different time points during the test cycle. At 6 h post-administration, there was no significant difference in serum T-AOC between the groups (*p* > 0.05). However, compared to the control group, the serum T-AOC of both the low-dose and high-dose groups decreased significantly 12 h after administration (*p* < 0.05). At 72 h post-administration, there was no significant difference in T-AOC in the low-dose group serum (*p* > 0.05), while the high-dose group serum showed a significant difference (*p* < 0.05).

Figure 1c illustrates the change trend of GSH-PX in serum at different time periods during the test cycle. After administration for 6–12 h, the content of GSH-PX in serum of both low-dose and high-dose groups decreased compared to the control group (*p* < 0.05). At 24 h after administration, the content of GSH-PX in the serum of the low-dose group was the lowest (*p* < 0.05), while the content in the serum of the high-dose group returned to the normal level (*p* > 0.05). Moreover, after 72 h to 7 days of administration, the content of GSH-PX in serum of both low-dose and high-dose groups was significantly higher than that in the control group (*p* < 0.05).

Figure 1d displays the changes in T-SOD content in serum during different time periods throughout the test cycle. No significant difference was observed in the serum T-SOD content between the low-dose and high-dose groups after 6–12 h of administration compared to the control group (*p* > 0.05). After 24 h of administration, a significant decrease in T-SOD content in serum was observed in the high-dose group (*p* < 0.05). Similarly, after 72 h of administration, a significant decrease in T-SOD content in serum was observed in the low-dose group (*p* < 0.05). However, after 7 days of administration, a significant increase in T-SOD content in serum was observed in both low-dose and high-dose groups (*p* < 0.05).

### 3.4 Effect of LPS combined with thrombin on immune indexes in a chick oxidative stress model

**Figure 2:**
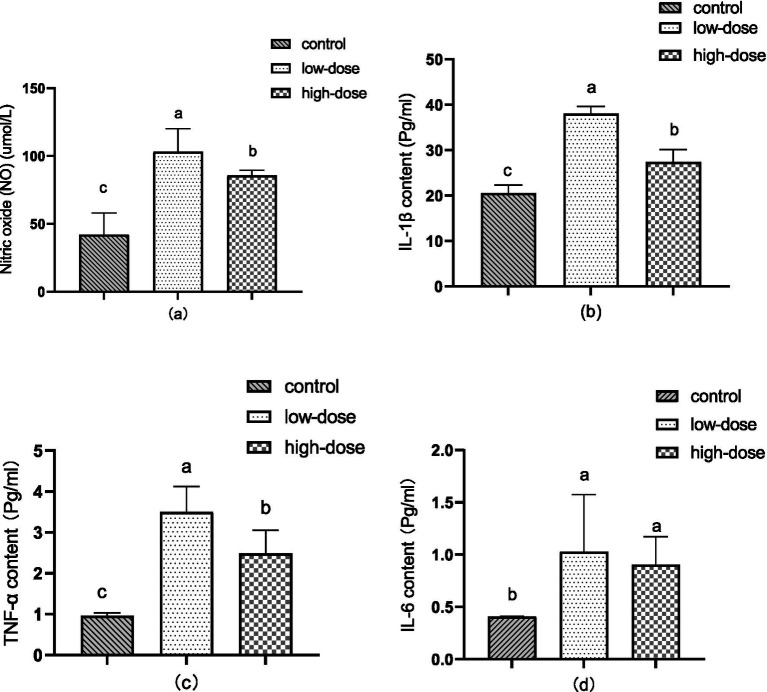
Effect of LPS combined with thrombin on immune indexes in a chick oxidative stress model (a) NO content (b) IL-1β levels (c) TNF-α content (d) IL-6 content.

Figure 2a illustrates the changes in serum nitric oxide (NO) content in the chick oxidative stress model induced by LPS and thrombin. After 12 h of administration, the content of NO in serum significantly increased (*p* < 0.05) compared to the control group, reaching 103.07 μmol/L in the low-dose group and 85.59 μmol/L in the high-dose group.

As shown in Figure 2b, the content of IL-1β in the liver increased significantly (*p* < 0.05) 12 h after administration compared to the control group. The low-dose group had a content of 38.01 pg/mL, while the high-dose group had a content of 27.37 pg/mL.

Showed in Figure 2c, the TNF-α content in the liver significantly increased (*p* < 0.05) after 12 h of administration compared to the control. The low-dose group had a TNF-α content of 3.50 pg/mL, while the high-dose group had a TNF-α content of 2.49 pg/mL.

In Figure 2d, compared to the control group, the content of IL-6 in the liver increased significantly (*p* < 0.05) after 12 h of administration, reaching 1.03 pg/mL in the low-dose group and 0.90 pg/mL in the high-dose group.

### 3.5 Histopathological observation of LPS combined with thrombin induced oxidative stress in chicks

#### 3.5.1 LPS combined with thrombin-induced liver oxidative stress in chicks

**Figure 3:**
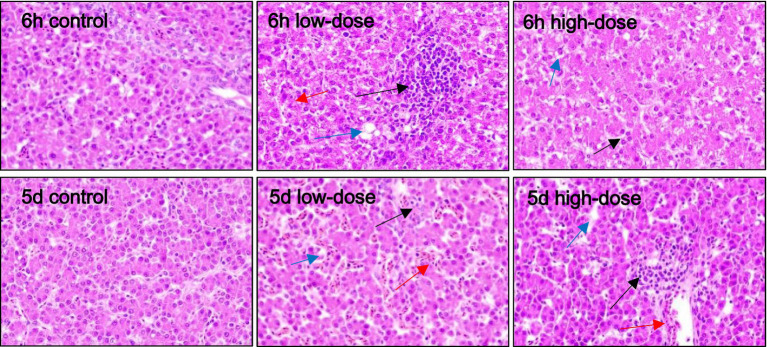
Pathological section observation of chick liver tissue (HE 400×). The hepatocytes are swollen, with vacuolar degeneration (blue arrows), dilated sinusoids, congestion and bleeding (red arrows), neutrophil infiltration, and varying degrees of inflammatory response (black arrows).

As shown in Figure 3, the results of HE staining showed that in the control group, liver cells were clear after injection for 6 h and 5 days. In the low-dose group, 6 h after injection, the liver cells exhibited massive vacuolar degeneration, with a slightly blurred boundary and infiltration of medium and fine granulocytes. After 5 days of injection, the sinusoid space expanded, with blood filling, liver cells swelling with vacuolar degeneration, and neutrophil infiltration. In the high-dose group, after 6 h of injection, the sinusoid space expanded, exhibiting slight hyperemia, hepatic cell vacuolar degeneration, and neutrophil infiltration. On the 5th day after injection, the liver cells swelled with vacuolar degeneration, the sinusoid space was slightly congested, and the vein was bleeding.

#### 3.5.2 Spleen of chick oxidative stress model induced by LPS and thrombin

**Figure 4:**
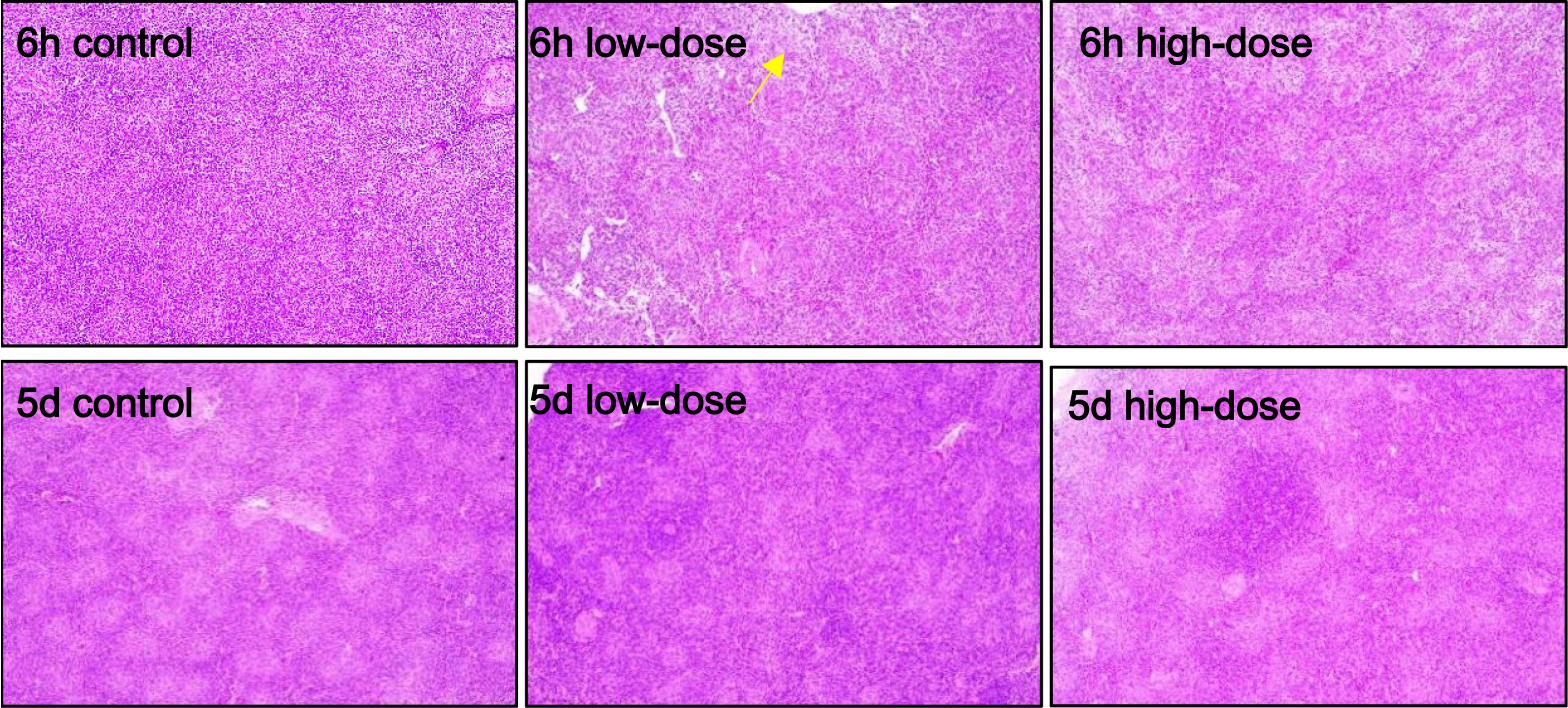
Pathological section observation of chick spleen tissue (HE 100×). There is necrosis of spleen parenchymal cells and reduction of white pulp (yellow arrow).

As shown in Figure 4, the spleen plays a crucial role in the immune system, and its response to LPS and thrombin-induced inflammation is important to understand. In the control group, the splenic cell morphology was normal. In the low dose group, there was no obvious abnormality after 5 days of injection. However, after 6 h of injection, the necrosis of spleen parenchyma cells and white pulp decreased significantly. In the high dose group, there was no obvious abnormality after 5 days of injection, and there was no significant change after 6 h of injection. These findings suggest that LPS and thrombin-induced oxidative stress had a limited impact on the spleen at the doses tested. Further studies are needed to investigate the effect of higher doses on spleen morphology and function.

#### 3.5.3 Oxidative stress of thymus in chicks induced by LPS combined with thrombin

**Figure 5:**
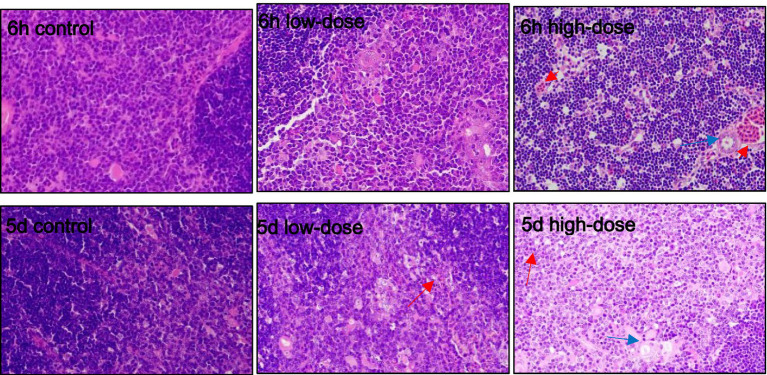
Pathological section observation of chick thymus tissue (HE 100×). The thymus showed varying degrees of erythrocytosis (red arrows), medullary thymocyte reduction, and vacuolar degeneration in the medullary region (blue arrows).

As shown in Figure 5, the control group showed no obvious abnormality in thymus morphology. In the low-dose group, varying degrees of erythrocytosis occurred at 5 days and 6 h after injection. In the high-dose group, a large number of erythrocytes proliferated in the thymus, and macrophages appeared in the cortex at 6 h after injection. The number of thymocytes in the medulla decreased, resulting in a pale color of the medulla. On the 5th day after injection, there was erythrocyte proliferation in the thymus, and a small amount of vacuolar degeneration appeared in the medulla.

#### 3.5.4 LPS combined with thrombin induced oxidative stress in chicks of intestinal segment

**Figure 6:**
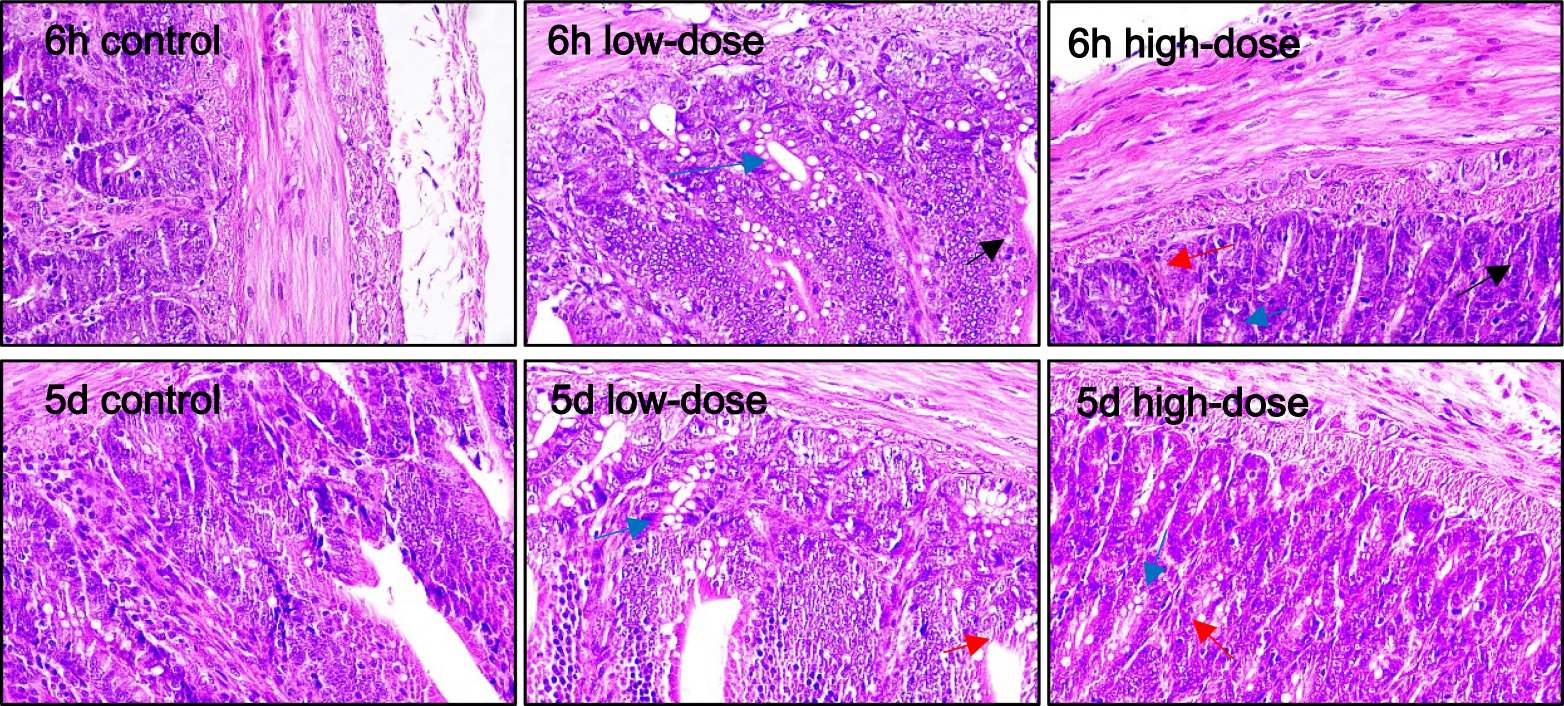
Pathological section observation of chick duodenum tissue (HE 40×). Duodenal histiocytes have vacuolar degeneration, morphological swelling (blue arrows), tissue damage, bleeding (red arrows), and lymphocyte infiltration has an inflammatory response (black arrows).

As shown in Figure 6, for duodenum, the morphology of histiocytes in the control group was normal at 6 h and 5 days after injection. In the low-dose group, there was obvious cell vacuolar degeneration and neutrophil infiltration at 6 h, and vacuoles still appeared at 5 days, with morphological changes and bleeding. In the high-dose group, there was a small amount of cellular vacuolar degeneration, neutrophil infiltration, tissue damage and bleeding at 6 h, and vacuolar and bleeding phenomena still existed at 5 days.

**Figure 7:**
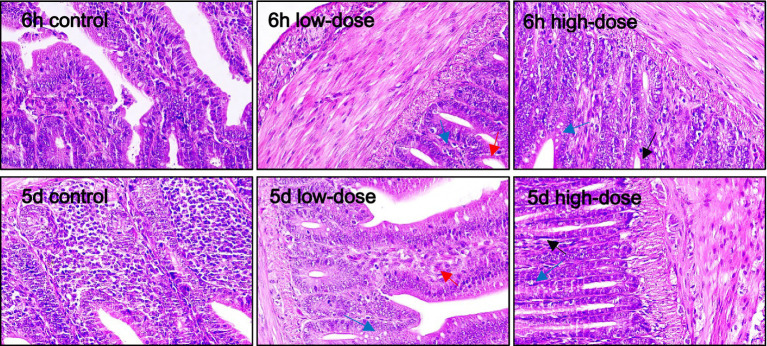
Pathological section observation of chick jejunum tissue (HE 40×). There is a small amount of vacuolar degeneration in the jejunal tissue cells (blue arrows), the cell morphology is blurred and occasionally hemorrhages (red arrows), and there are some aggregations of neutrophils (black arrows).

As shown in Figure 7, the control group had normal jejunal cell tissue morphology. The low-dose group had a small amount of vacuoles and oozing blood 6 h and 5 days after injection. In the high-dose group, neutrophils were infiltrated after 6 h and 5 days, and inflammation appeared, and some vacuoles could be observed.

**Figure 8:**
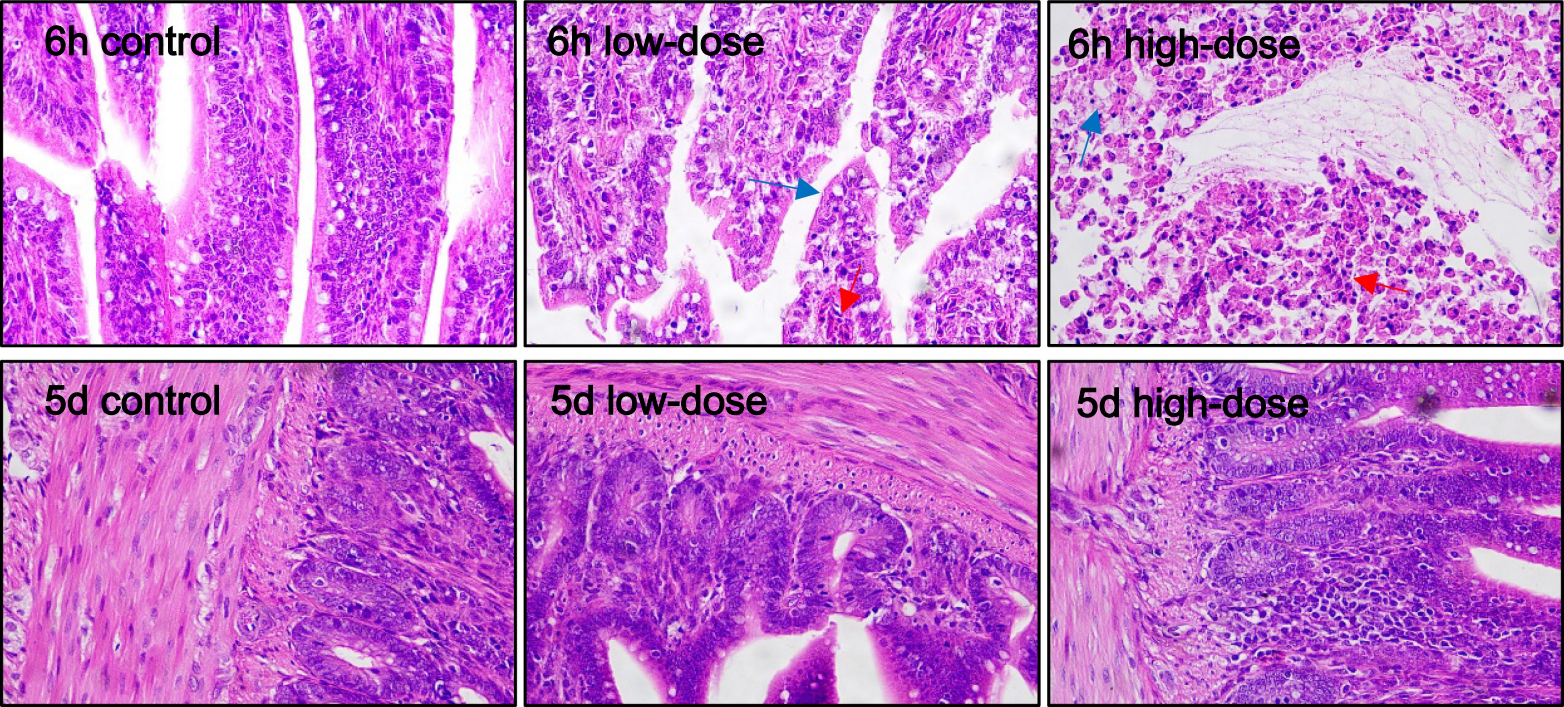
Pathological section observation of chick ileum tissue (HE 40×). Ambiguous ileal tissue cell boundaries with oozing blood (red arrows), cell morphological changes, vacuolar degeneration (blue arrows).

As shown in Figure 8, at 6 h, the low-dose group and the high-dose group of ileal histiocytes showed obvious cell morphological changes, vacuolar degeneration, obvious damage and bleeding.

## 4 Discussion

### 4.1 The effect of LPS combined with thrombin on chick oxidative stress model’s temperature

LPS is capable of inducing the expression and secretion of inflammatory cytokines such as TNF-α through various signaling pathways, including NF-κB and MAPK, ultimately triggering inflammation in the organism (14). Furthermore, LPS serves as an exogenous pyrogen by stimulating immune cells within the organism to produce endogenous pyrogens (TNF-α, IL-1β). These endogenous pyrogens elicit fever by stimulating mediators in cortical cells of the brain or directly participating in the synthesis and release of PGE2, a central mediator of fever (15, 16). Through studies of nitric oxide synthase inhibition and endotoxin-induced fever L-CIT on body temperature and inflammatory responses, LPS-induced hyperthermia was found to be associated with increased concentrations of NO and PGE2 (17). Additionally, literature suggest that LPS stimulation in chicks can easily cause endotoxemia in their intestines, leading to a fever response (18). It was also shown that the reduction of body temperature during systemic inflammation in chicks included modulation of the hypothermic response to LPS, inhibition of thermogenesis with metabolic inhibition (19). Therefore, in the early stages of the experiment, chick rectal temperature was used as a basis to explore the early symptoms and clinical phenomena of the chick oxidative stress model. Experimental results showed that using LPS combined with thrombin to stimulate 4-day-old chicks could cause changes in their rectal temperature. The low-dose group’s rectal temperature began to drop to normal after 18 h, while the high-dose group continued to exhibit a fever response. Chick clinical symptoms included depression, decreased food intake, and some chicks clustering and sleeping, but these clinical symptoms were resolved or disappeared after a certain period of self-regulation.

### 4.2 Impact of LPS and thrombin combination on growth performance in a chick oxidative stress model

Prior research has demonstrated that exposure to LPS impairs both the innate and adaptive immune systems of chicks, eliciting an inflammatory response and subsequently compromising growth performance (20, 21). Furthermore, LPS-induced systemic inflammation has been shown to adversely affect bone formation in chicks by inhibiting processes such as bone modeling, remodeling, and extracellular matrix functions (22). In this study, the synergistic effect of LPS and thrombin significantly decreased daily weight gain and worsened the feed conversion ratio in chicks. This phenomenon can be attributed to the combined activation of the hypothalamic–pituitary-adrenal axis and the disruption of appetite signaling receptors by LPS and thrombin, ultimately inducing anorexia (23). Additionally, the concurrent administration of LPS and thrombin prompts the body to produce a substantial quantity of pro-inflammatory cytokines. Consequently, nutrients from the feed are redirected towards the immune system, resulting in decreased nutrient utilization efficiency and further diminished growth performance in chicks (24).

### 4.3 Effect of LPS combined with thrombin on serum indicators in a chick oxidative stress model

LPS has the capability to elicit inflammation, oxidative stress, and apoptosis in bovine mammary epithelial cells. This induction results in the excessive production of ROS and MDA, while concurrently diminishing the activity of antioxidant enzymes, such as SOD, and suppressing the expression of HO-1 protein (25). The accumulation of excessive ROS is detrimental to macromolecules, including proteins and DNA, as well as to mitochondrial function, ultimately causing cellular and tissue damage (26, 27). Accumulating evidence proposes a bidirectional relationship where inflammation initiates oxidative stress, which subsequently amplifies the inflammatory response (28–30). The experimental findings demonstrate that the combined exposure to LPS and thrombin markedly elevates MDA levels while reducing the activity of serum T-AOC, GSH-PX, and SOD. These observations suggest that the synergistic stress induced by LPS and thrombin disrupts the normal balance of the oxidative/antioxidative system *in vivo*, leading to the manifestation of oxidative stress.

### 4.4 LPS combined with thrombin-induced chick oxidative stress model: impact on liver immune indexes

Interleukin is a type of cytokine produced by various cells, which plays a critical role in the immune function of the human body, including cell proliferation and immune regulation. When bacteria or pathogens enter the body, the secretion of pro-inflammatory cytokines (IL-1β, IL-6, TNF-α) in the body can lead to immune responses of cells and tissues (31). Research has shown that LPS produced by G-bacteria is the main cause of organ damage in the body, and the first target organ attacked by LPS is the liver (32). This stimulation activates downstream NF-κB signaling pathway and regulates mitogen-activated protein kinase to cause a series of immune responses in the body (33), ultimately leading to liver cell damage. Studies have reported that LPS can alter the liver microenvironment by regulating the inflammatory mediators related to M1 or M2 macrophages and macrophage hepatotoxicity (34). The combination of cefoperazone sodium with LPS significantly increased the content of IL-1β, IL-6, and TNF-α pro-inflammatory cytokines in the serum of broiler chicks and induced chick liver damage (35, 36). The results of this experiment showed that compared with the control group, the low-dose and high-dose groups showed an increase in the content of IL-1β, IL-6, and TNF-α pro-inflammatory cytokines in the liver. However, the level of pro-inflammatory cytokines was low, which may be due to the fact that the experiment only extracted the inflammatory factors in the liver tissue and could not reflect the expression level of systemic inflammation. As the liver belongs to the glandular epithelial cells and the digestive tract epithelial cell system, the body must have anti-inflammatory responses to counteract excessive inflammatory responses and reduce the damage caused by excessive inflammation to the body.

### 4.5 The effects of combined LPS and thrombin on tissue pathology in a chick oxidative stress model

The bursa of Fabricius and thymus are central lymphoid organs in chicks that are critical for the development of adaptive immunity (37). Thymic cells release various cytokines, including IL-1, IL-3, IL-4, and IL-6, as well as hormones such as thymopoietin and thymosin, which play important roles in regulating immune responses (38). Research has shown that inducing inflammation through LPS stimulation can lead to tissue damage, vascular reactions, and cell proliferation (39). Pathologically, LPS stimulation results in the appearance of vacuoles in tissue cells, which is known as “ballooning.” During the development of inflammation, cell proliferation becomes increasingly apparent in the later stages. Mechanistically, studies have indicated that certain cytokines produced during inflammation can stimulate cell proliferation (40). LPS stimulation also results in varying degrees of tissue hemorrhaging.

Poultry have a certain tolerance to LPS, and single stimulation of poultry with LPS alone cannot cause obvious infection (41). However, with the assistance of thrombin, an oxidative stress model in poultry can be established. The results of this experiment showed that under the injection of LPS combined with thrombin, by collecting chick liver, spleen, intestine, and thymus tissues and making slices, varying degrees of erythrocyte proliferation and tissue cell vacuolar degeneration can be observed in chick tissues. Through the experiment, it was found that LPS combined with thrombin can induce oxidative stress responses in chicks, and the degree of pathological tissue damage in chicks varies at different concentrations. The tissue damage in chicks was more significant under the stimulation of low concentrations of LPS combined with thrombin.

According to the literature, the use of LPS alone to establish an inflammatory model in poultry is not ideal, possibly due to insufficient clotting factors in poultry. Some studies have found that combined effects when LPS is used for inflammation induction (42). Other research has found that the clotting enzyme in the white-lipped pit viper is composed of prothrombin and thrombin, and their mechanisms of action are similar. Under the action of Ca^2+^, they can activate factors V, VII, VIII, and promote platelet aggregation. Prothrombin, under the action of platelet factor III, can transform prothrombin into thrombin, activate factor V, and affect factor X (43). At the same time, research has shown that injection of low-dose white-lipped pit viper venom clotting enzyme has a pro-coagulant effect in the body, while high-dose injection has an anticoagulant effect (44). Under low-dose action, the chick coagulation system is activated, thus activating clotting factors. Under high-dose action, the chick coagulation system is inhibited, resulting in the inhibition of clotting factors (45). In this experiment, chicks were stimulated with LPS combined with thrombin. The results showed that under low-dose action, the chick oxidative stress response was significant, while under high-dose action, the chick oxidative stress response was not significant. The reason may be that the high dose of thrombin inhibits the coagulation system. Therefore, the low dose of thrombin and LPS used in this experiment is the most suitable concentration for constructing a chick oxidative stress model.

## 5 Conclusion

This study utilized 4-day-old AA broiler chicks to investigate the influence of different concentrations of LPS combined with thrombin on the growth performance, serum antioxidant indexes, and immune indexes of broiler chickens. Based on the findings of the present study, it can be concluded both the low-dose and high-dose groups can trigger the oxidative stress response of chicks, and the oxidative stress response of the low-dose group is more pronounced, and therefore the low-dose group in this study is the most suitable concentration to constitute an effective experimental model of chick oxidative stress.

## Data Availability

The original contributions presented in the study are included in the article/supplementary material, further inquiries can be directed to the corresponding author.
